# Phylogeography of *Hyalomma* (*Euhyalomma*) *lusitanicum* (Acarina, Parasitiformes, Ixodidae) in Andalusia based on mitochondrial cytochrome oxidase I gene

**DOI:** 10.1007/s10493-021-00652-0

**Published:** 2021-08-25

**Authors:** Francisco J. Márquez, Antonio Caruz

**Affiliations:** 1 Departamento de Biología Animal, Biología Vegetal y Ecología, Universidad de Jaén, Campus Las Lagunillas, s.n., 23071 Jaén, Spain; 2 Departamento de Biología Experimental, Universidad de Jaén, Campus Las Lagunillas, s.n., 23071 Jaén, Spain

**Keywords:** *Hyalomma lusitanicum*, Phylogeography, COI gene, Andalusia

## Abstract

The genetic population structure relationships of *Hyalomma* (*Euhyalomma*) *lusitanicum* in Andalusia (the south of the Iberian Peninsula) were examined using mtDNA sequence data from 887 bp of cytochrome oxidase subunit I (COI) gene. The sequence for the COI region was determined for 84 individuals collected in several localities of Andalusia, and 10 for other localities (i.e., five from Toledo, central Iberian Peninsula, four from Sicily (Italy) and one from Canary Island). Seventeen haplotypes were detected, including 27 polymorphic sites. The number of amino acid substitutions per site from mean diversity calculations for the entire population was 0.017. AMOVA analysis revealed a low gene flow that characterises the genetic population structure of this species in South Iberian Peninsula, with a haplotype diversity (h) value of 0.815. No geographically induced differentiation was observed, and separate evolutionary units were not detected. Our results indicate low genetic diversity across the geographical range of *H. lusitanicum* tick in Andalusia. Our data do not show any genetic discontinuity between the tick populations studied, including specimens from Canary Island and Sicily (Italy).

## Introduction

*Hyalomma lusitanicum* Koch has been included in the ‘*anatolicum*’ group, together with several species and subspecies (*H. anatolicum, H. excavatum* and *H. franchinii*) (Guglielmone et al. 2014). The mature phases of this species, like all other types of subgenus *Euhyalomma*, parasitize large ungulates, mainly cattle (Apanaskevich 2004). On the Iberian Peninsula, its distribution range is restricted to the southern half (Estrada-Peña et al. 2004; Estrada-Peña and Santos-Silva 2005; Ruiz-Fons et al. 2006). It is also known to occur on the Balearic Island of Menorca (Castellà et al. 2001). Furthermore, the species has been reported from Morocco, Algeria, Italy (including Sicily) and France (Torina et al. 2006, 2010; Santos-Silva 2017). Due to the strategic geographical position, Andalusian *H. lusitanicum* populations represent a melting pot that connect other Iberian Peninsula populations with North Africa populations as well as with Mediterranean and Atlantic colonized islands with dispersion driven by bird migration and human action (Palomar et al. 2013).

The three-host life cycle of *H. lusitanicum* has been studied under laboratory conditions (Ouhelli and Pandey 1984; Ouhelli 1994), with larva and nymph stages able to feed on rabbits and cattle, whereas adults engorge on cattle but not on rabbits. Such studies have not been conducted under natural conditions, but the Mediterranean rabbit and various ungulates may be the main hosts for immature and adult *H. lusitanicum* ticks, respectively (González et al. 2016; Valcárcel et al. 2016).

Several *Hyalomma* species are susceptible to infection with the highly pathogenic *Theileria annulata*, the causative agent of tropical theilerosis (Sayin et al. 2003). In the Iberian Peninsula and the Balearic Islands, *T. annulata* coexists frequently with *T. buffeli* which is considered as a non-malignan species (Georges et al. 2001; Brígido et al. 2004), likely transmitted by *Haemaphysalis* spp. ticks (García-Sanmartín et al. 2006). In this region, both *H. lusitanicum* and *H. marginatum* has been incriminated in the transmission of *T. annulata* and *Theileria* spp. (Habela et al. 1999; Viseras et al. 1999; Almería et al. 2001; Santos-Silva et al. 2011; Gomes et al. 2013; Pereira et al. 2016). In recent times, *H. lusitanicum* has been implicated as reservoir of Crimean-Congo haemorrhagic fever virus (CCHFV) in central Iberian Peninsula (Estrada-Peña et al. 2012a, b; Negredo et al. 2019).

The wide distribution of CCHFV (genotype Africa IV and Europe V) in the centre and southwest of the Iberian Peninsula in various species of ungulates (red deer, fallow deer and Eurasian wild boar) and associated tick species *H. lusitanicum* and *D. marginatus* (Moraga-Fernández et al. 2020) introduces the need to supplement epidemiological studies with studies that highlight the phylogeography of potential vector ticks of this arbovirus in order to establish their population structure and possible dispersal routes in this area. The phylogeny of ticks has been extensively studied to determine the relative positions of genera (Murrell et al. 2001). Mitochondrial genes are among the most widely used markers for phylogenetic studies of animals (Avise 2004). In the case of ticks, mitochondrial DNA (mtDNA) sequences have been extensively used to investigate between populations within species (Mtambo et al. 2007) and the relationships of closely related species (Caporale et al. 1995; Zahler et al. 1995) for their rapid rate of evolution.

Molecular studies focussing on *Hyalomma* genus are very scarce and partial in the taxonomic and geographical scopes. More recently, the use of mtDNA sequences to study the phylogenetic relationships and evolutionary history of Eurasian and African *Hyalomma* species has been advocated (Rees et al. 2003; Sands et al. 2017; Roth et al. 2019). The main objective of this work is to evaluate mtDNA cytochrome oxidase subunit (COI) gene diversity in *H. lusitanicum* populations to examine the biogeographic and phylogenetic relationships and determine the coherence of evolutionary lineages in Andalusia. This gene has focused increasing attention when it is used as a standardized DNA region to diagnose and delimit species by DNA barcoding (Hebert et al. 2003; Roe and Sperling 2007; Zhang and Zhang 2014).

## Material and methods

### Samples and DNA extraction

**Fig. 1:**
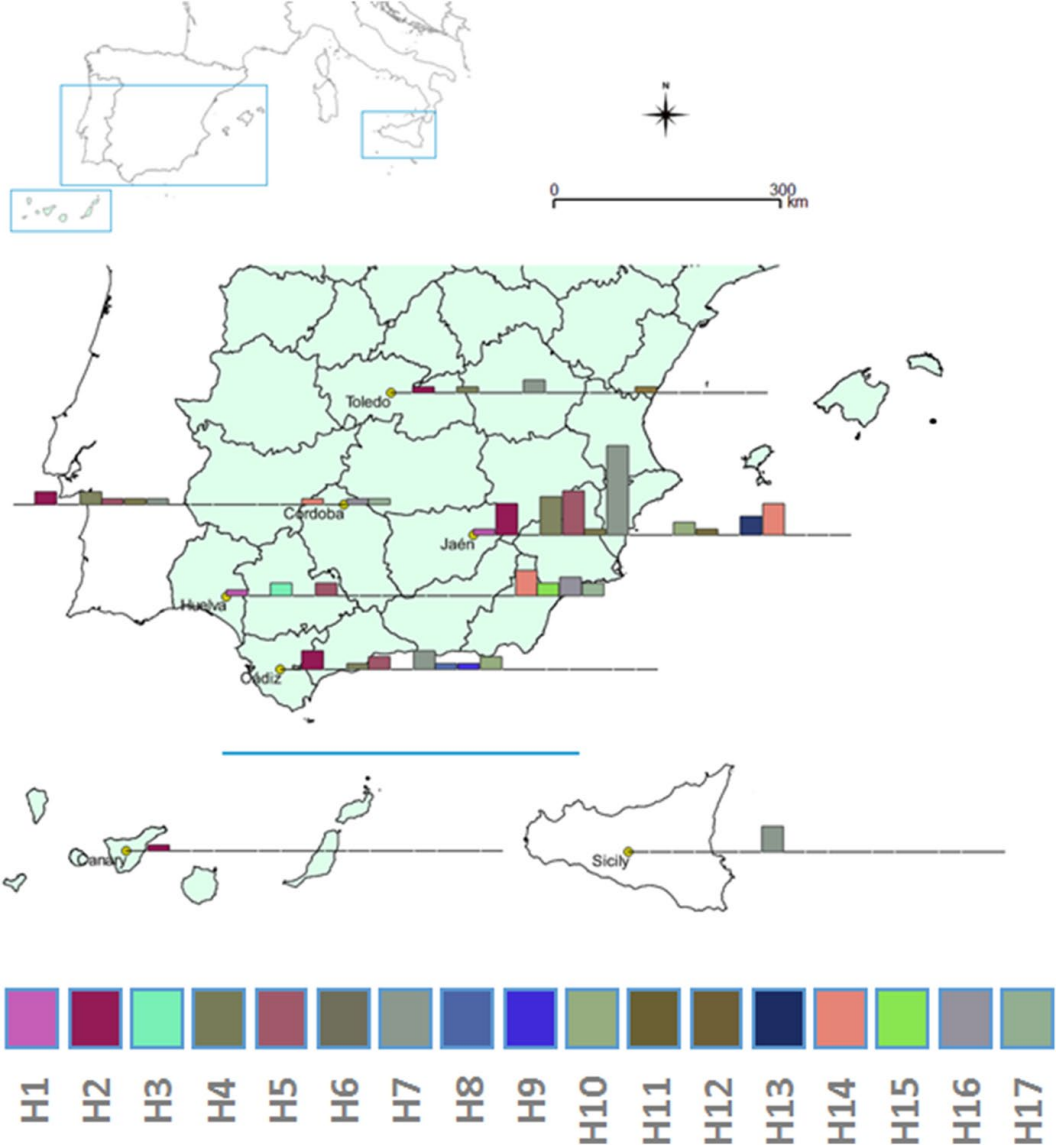
Map of southern Spain, Canary Island and Sicily (Italy) indicating sampled localities and the haplotype frequency in each of the sites considered

In total, 84 *H. lusitanicum* adult ticks were collected on vegetation by dragging in Andalusia, and 10 from other localities (Toledo, central Iberian Peninsula; Gran Canaria, Canary Island; and Sicily, Italy) (Fig. 1), mainly in summer, when adults of this species have the highest activity (Requena-García et al. 2017). After collection, the ticks were immediately placed in vials with 70% ethanol and properly labelled. Identification was confirmed in the laboratory based on taxonomic keys (Gil Collado et al. 1979; Manilla and Giannetto 1996; Apanaskevich et al. 2008).

Ticks were kept individually, rinsed with distilled water, dried on sterile filter paper and then crushed in sterile Eppendorf tubes. DNA was extracted using a DNA tissue kit according to the manufacturer’s instructions (Macherey–Nagel, Düren, Germany). During DNA extraction, we included sterile water as a negative control using a ratio of one control for every 15 samples. DNA extracts were stored at −20 °C until further processing.

### Polymerase chain reaction (PCR)

A 887-bp fragment of the cytochrome oxidase subunit I (COI) gene was amplified and sequenced for each specimen. PCR amplifications were carried out in a MJ Mini Personal Thermal Cycler (Biorad, Hercules, CA, USA) using the primers hylCOIfor 5′-AATTTACAGTTTATCGCCT and HylCOIrev 5′-CATACAATAAAGCCTAATA (MWG, Ebersberg, Germany). Each PCR mixture consisted of the following: 4 μL of DNA, 20 pmol of each primer, 200 µM of dATP, dCTP, dTTP, dGTP, 2.0 mM MgCl_2_, 0.033 U of Biotaq DNA polymerase in 1 × PCR ammonium buffer (Bioline, Randolph, MA, USA), and sterile distilled water to a final volume of 80 μL. PCR cycles included an initial 90 s denaturation step at 96 °C, followed by 35 cycles of denaturation at 94 °C for 30 s, annealing at 48 °C for 30 s, and extension at 72 °C for 50 s. Amplification was completed by holding the reaction mixture at 70 °C for 7 min to allow complete extension. PCR products were resolved by electrophoresis in 1.5% SeaKem agarose (Cambrex, Rockland, ME, USA) in 1 × buffer Bionic gels (Sigma, St. Louis, MO, USA) using a 100 bp ladder as molecular weight marker (Eurogentec, Seraing, Belgium). Products containing positive results were purified by using the Montage PCR kit (Millipore, Bedford, MA, USA) prior to sequencing. Negative controls were further processed by PCR as tick specimens. In order to reduce the risk of contamination no positive controls were used.

For the purpose of checking for potential amplification of nuclear mitochondrial paralogs (numts) (Moulton et al. 2010; Calvignac et al. 2011), we used two in silico strategies. First, the primers were aligned with the reference genome of *Drosophila melanogaster* using the UCSC (https://genome.ucsc.edu/cgi-bin/hgBlat)—both the forward and reverse primers did not display homology with any nuclear sequence. Second, the software Blast Primer (https://www.ncbi.nlm.nih.gov/tools/primer-blast) did not predict any amplification from the *Ixodes scapularis* nuclear genome.

### Sequencing and sequence analysis

Positive PCR products were sequenced using PCR primers and the GenomeLab DTCS-Quick Start kit (Beckman Coulter, Fullerton, CA, USA) and a CEQ 2000XL capillary DNA sequencer (Beckman Coulter) according to the manufacturer’s instructions. In addition to forward and reverse primers two internal primers (HylCOIfor2 5′-GGATAACAATAGAACGTATAC and HylCOIrev2 5′-CAGTTCCTGCTCCTGATC) were used to read terminal regions. The forward and reverse sequences obtained were assembled into contigs with the program Bioedit v.7.0.1 (Hall 1999). The resulting COI sequences were manually aligned and analysed to obtain consensus sequences and to align and compare with other tick sequences found on GenBank database, using the BLAST feature (http://ncbi.nlm.nih.gov/blastn) (Altschul et al. 1990).

### Phylogenetic analyses

The COI gene region was nearly length invariant for all the samples, making alignment trivial. Nucleotide variation and substitution patterns were examined using the software package MEGA7 (www.megasoftware.net; Kumar et al. 2016). Standard genetic indices, haplotype diversity (h) and nucleotide diversity (π), were computed.

Phylogenetic relationships between haplotypes were inferred by constructing webs connecting haplotypes of *H. lusitanicum* using the complete dataset using Median-joining and Templeton–Crandall–Sing (TCS) networks algorithms (Templeton et al. 1992; Clement et al. 2000), available in PopArt (http://popart.otago.ac.nz) (Bandelt et al. 1999). This program determines the number of polymorphic sites, h, π and the average number of nucleotide differences.

Molecular diversity indices were generated for seven populations and each gene using Arlequin (Excoffier and Lischer 2010). The population genetic structure and neutrality of populations were tested by analysis of molecular variance (AMOVA), including the overall fixation index statistics (FST) and pairwise FST with 1023 permutations.

## Results

Nucleotide sequence of the COI region was determined for all 94 *H. lusitanicum* samples. Mitochondrial DNA polymorphism in the fragments showed 27 polymorphic sites that differentiated 17 distinct haplotypes (Fig. 1; GenBank accession codes and localities are presented in Table 1). The observed nucleotide frequencies were 0.295 (A), 0.385 (T/U), 0.173 (C), and 0.146 (G). The number of amino acid substitutions per site from mean diversity calculations for the entire population was 0.017.

**Table 1:** GenBank accession numbers of the 17 haplotype sequences and representation in the seven sampled populations

Haplotype	GenBank	Cádiz	Córdoba	Huelva	Jaén	Toledo	Sicily	Canary Island	No. populations
1	EU827697	0	0	1	1	0	0	0	2
2	EU827698	3	2	0	5	1	0	1	5
3	EU827709	0	0	2	0	0	0	0	1
4	EU827699	1	2	0	6	1	0	0	4
5	EU827712	2	1	2	7	0	0	0	4
6	EU827710	0	1	0	1	0	0	0	2
7	EU827719	3	1	0	14	2	4	0	5
8	EU827725	1	0	0	0	0	0	0	1
9	EU827724	1	0	0	0	0	0	0	1
10	EU827722	2	0	0	2	0	0	0	2
11	EU827731	0	0	0	1	0	0	0	1
12	EU827734	0	0	0	0	1	0	0	1
13	EU827720	0	0	0	3	0	0	0	1
14	EU827735	0	1	4	5	0	0	0	3
15	EU827737	0	0	2	0	0	0	0	1
16	EU827743	0	1	3	0	0	0	0	2
17	EU827741	0	1	2	0	0	0	0	2

For our dataset, MEGA7 suggested the T92+G model (Tamura 3-parameters; Tamura and Nei 1993). Given that the trees were best resolved, the maximum likelihood (ML) and neighbor joining (NJ) analysis were based on this model (value of –lnL = –1382.581; BIC = 3092.278; AICc = 2833.320 and gamma shape = 0.43).

**Fig. 2:**
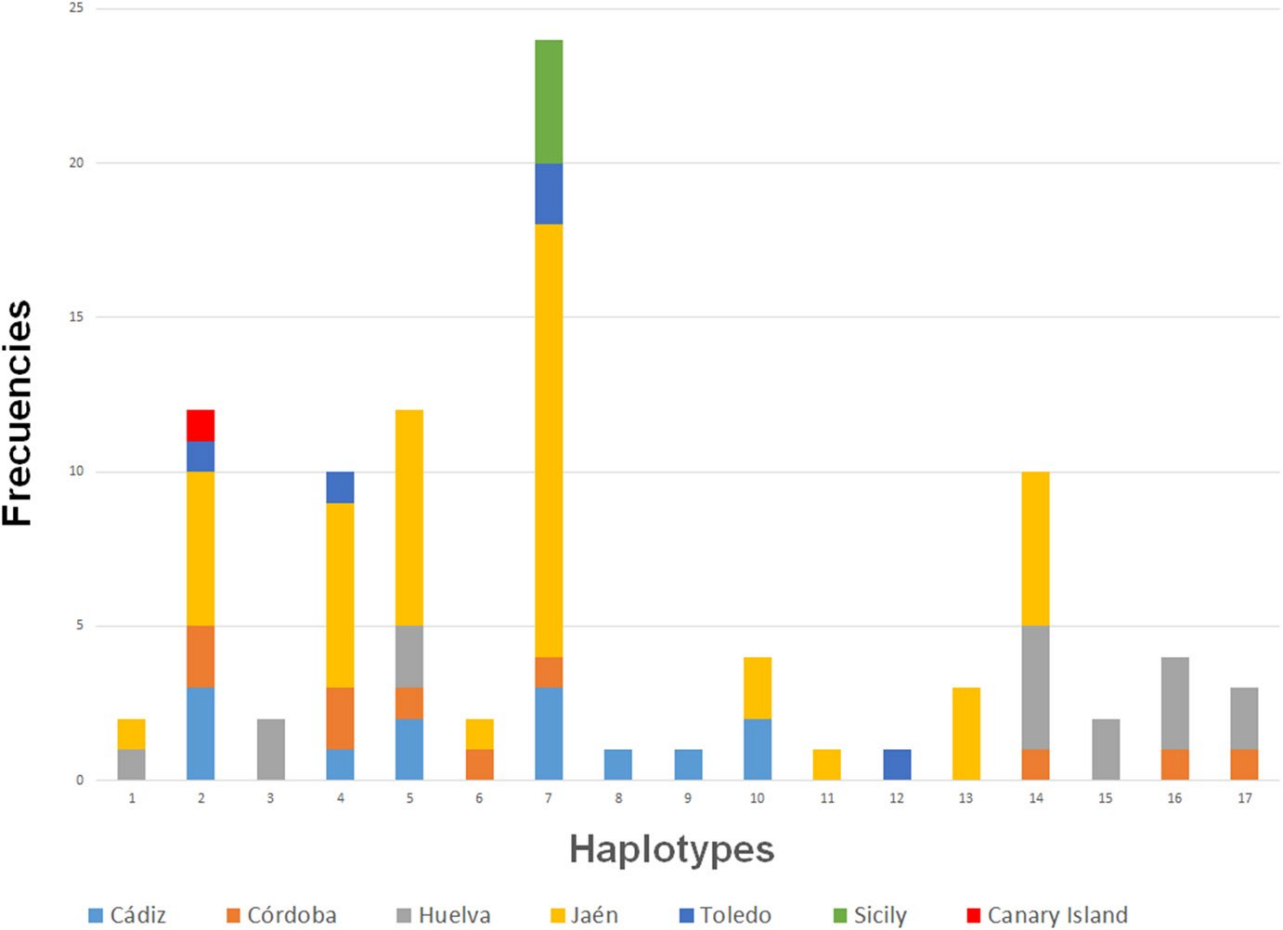
COI haplotype frequencies for the seven sites considered in this study. The Andalusian sites are Cádiz, Córdoba, Huelva and Jaén

**Fig. 3:**
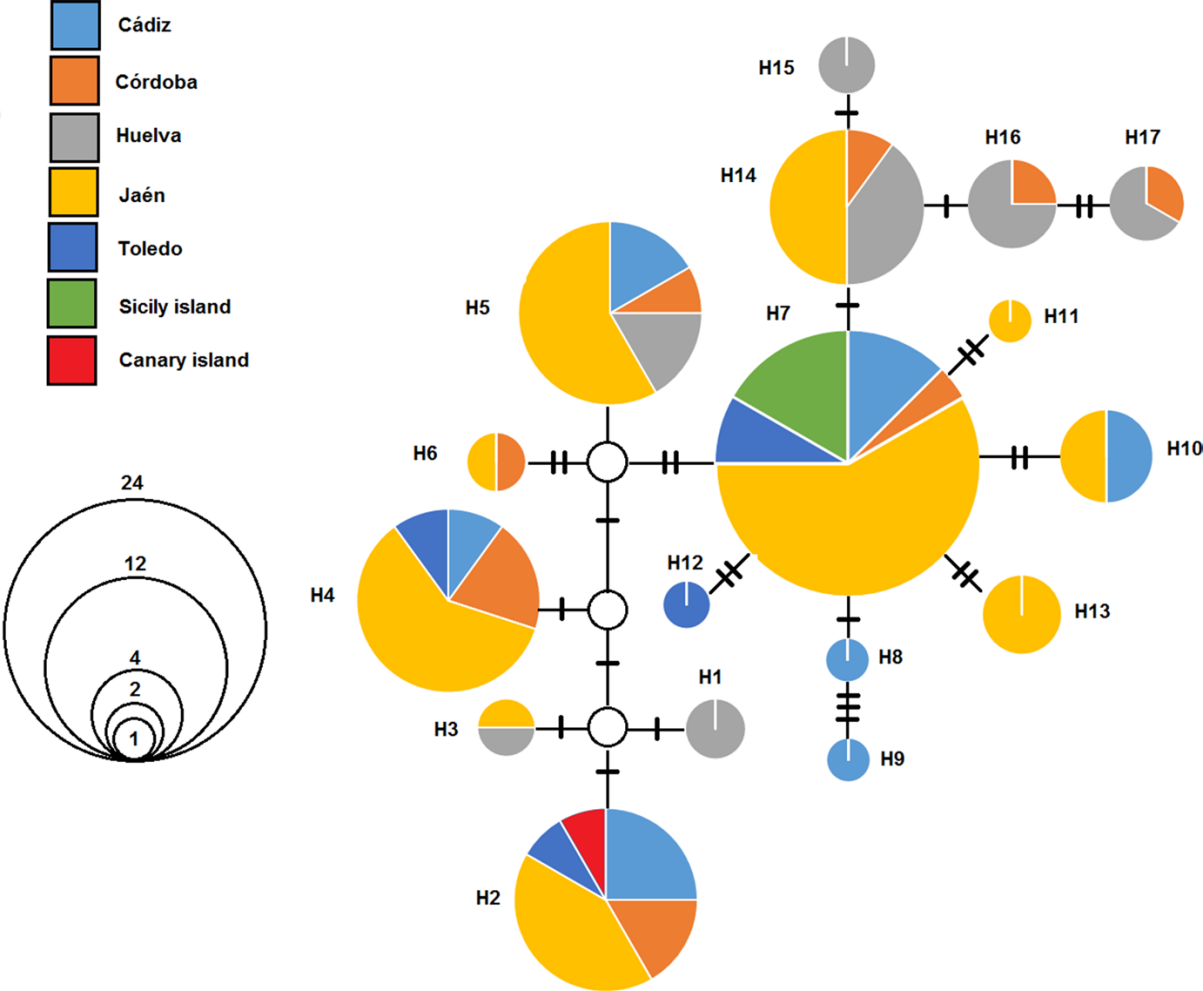
Nested clade design in the minimum spanning tree for *Hyalomma lusitanicum* mitochondrial haplotypes. Circle size is proportional to the number of similar haplotypes observed in the dataset. Small white circles indicate missing haplotypes. The number of bars between two circles represents the number of mutations between haplotypes

The parsimony analysis between haplotypes indicated the existence of 860 constant characters. Among the 27 variable characters, seven were parsimony-informative and 20 were singleton sites. The unweighted parsimony search resulted in one phylogenetic tree. Maximum parsimony of the unweighted data yielded a tree length of 28 steps along with corresponding bootstrap percentages. The consistency index (CI) for the tree was 0.875 and the retention index (RI) 0.952. The frequencies obtained for each haplotype and the population of origin are represented in Fig. 2, the minimum spanning network is shown in Fig. 3. Nucleotide diversity (π) was 0.004, with seven parsimony-informative sites. Tajima D statistic has a calculated value of −0.963, whereas p (D ≤  −0.963) was 0.824.

Because of unequal sample sizes, it is not always possible to compare the genetic diversity obtained from distinct geographical regions. Higher genetic variability is expected in source populations. Haplotypes 2 and 7 were present in four of the Iberian Peninsula sites plus in Canary Island (ht 2) and Sicily (ht 7) (Table 1). Seven haplotypes (3, 8, 9, 11, 12, 13 and 15) were found in only one site. In Andalusia, 16 of the 17 haplotypes identified in this study were found; only haplotype 12 appeared exclusively in a population outside the area (Toledo; Table 1, Fig. 2). Ten haplotypes appeared in the widely sampled and easternmost province of Jaén. Six other haplotypes detected in Andalusia were found in Jaén (Figs. 1 and 2).

The AMOVA analysis (Table 2) indicated that the COI gene exhibited high and significant differentiation among populations (FST = 0.053, P < 0.01). Most of the genetic variation (94.7%) was explained by differences among individuals within a population, whereas the remainder of the variation was attributable to differences among populations. Haplotype diversity (h) ranged from 0.720 (Toledo) to 0.860 (Córdoba), the mean value for the Iberian Peninsula populations was 0.815 (Table 3). Considering only Iberian ticks, 80% of the Shannon information index (^s^H) is due to differences within the five sites considered.

**Table 2:** AMOVA results for the COI sequences of studied populations

Source of variation	d.f	SS	Variance components	% variation
Among populations	6	20.29	0.12	5.33
Within populations	87	186.99	2.15	94.67
Total	93	207.94	2.27	

FST = 0.053, P < 0.01

**Table 3:** Molecular diversity indices of mitochondrial DNA across seven populations

Population	Sample size	No. haplotypes (Na)	No. effective haplotypes (Ne)	Information index (I)	Haplotype diversity (h)	Unbiased haplotype diversity (uh)
Cádiz	13	7	5.828	1.845	0.829	0.898
Córdoba	10	8	7.143	2.026	0.860	0.956
Huelva	16	7	6.096	1.874	0.836	0.892
Jaén	45	10	5.836	1.983	0.829	0.848
Toledo	5	4	3.572	1.333	0.720	0.900
Mean ± SE	17.8 ± 7.0	7.2 ± 0.97	5.695 ± 0.584	1.812 ± 0.125	0.815 ± 0.025	0.899 ± 0.018
Sicily	4	1	1	–	–	–
Canary Island	1	1	1	–	–	–

The graph representing the phylogenetic network visualizes population relationships defined by haplotypes separated by a maximum of 13 mutations (Fig. 3).

## Discussion

Our mitochondrial data indicate low genetic diversity across the geographical range of *H. lusitanicum* in Andalusia. No genetic discontinuity is apparent between the tick populations, including specimens from Canary Island and Sicily.

Sands et al. (2017) show a phylogeny of *Hyalomma* ticks based on their evolutionary history in relation with tectonic events and large-scale environmental changes registered during a long period, with a divergence time around 10 million years ago. In this context, *H. lusitanicum* occupied a marginal area of distribution in the Iberian Peninsula and a scarce number of historically related island territories. The presence of this species out of this area is mainly anecdotic (Santos-Silva 2017). The low COI genetic diversity observed in Canary Islands and Sicily may be due to founder events and the maternal inheritance of mtDNA, but this hypothesis has to be confirmed using greater sample sizes. The results indicated that gene flow of the maternal lineage is relatively broad.

The distribution of *H. lusitanicum* haplotypes is the result of a long interaction of this species with its natural hosts. However, the current distribution of the various haplotypes has been influenced by anthropogenic action, manifested as habitat modification and the introduction of new host species through domestication (Hoberg and Brooks 2008; Ledger and Mitchell 2019). The movement of large masses of livestock (especially cattle, goats, sheep and horses) related to transhumance must have affected the phylogeography of the species in a relatively small area over time. This activity has been actively developed in the Iberian Peninsula over many centuries. Animal herders have used well-known travel routes, initially through the Roman roads and more recently through the glens, from the 13th century onwards (Walker 1983). This determined seasonal migrations of livestock from North to South and vice versa in a fall-spring cycle, concerning ovine, bovine, caprine and equine herds (Gómez Sal and Lorente 2004) that permit the gene flow among populations and haplotypes widespread in the distribution area of *H. lusitanicum*. Migratory movements also represent a unique dispersal mechanism (Bauer and Hoyer 2014), and long-distance dispersal events may be highly important for the re-colonization of unoccupied habitats, the recovery of lost populations and maintenance of gene flow (Viana et al. 2013). Bioclimatic conditions limit the actual distribution of *Hyalomma* species, as has been hypothesized by Estrada-Peña et al. (2011, 2012a, b) for *H. marginatum*. Wild fauna displacements, megafauna disappearance in southwest Europe (Malhi et al. 2016) and transhumance in recent times have contributed to the formation of a valuable set of sylvopastoral landscapes and associated natural habitats (Gómez Sal and Lorente 2004). Changes in the ecology of migratory species could have enormous impact on pathogen spread in wildlife and livestock, as well as altering human exposure to zoonotic infections (Hoberg et al. 2008; Altizer et al. 2011). Further investigation of the phylogeographic history of this species would be useful, increasing the sample size from various parts of its distribution area and increasing the number of genetic markers.
